# Muscle regeneration affects Adeno Associated Virus 1 mediated transgene transcription

**DOI:** 10.1038/s41598-022-13405-9

**Published:** 2022-06-11

**Authors:** Amédée Mollard, Cécile Peccate, Anne Forand, Julie Chassagne, Laura Julien, Pierre Meunier, Zoheir Guesmia, Thibaut Marais, Marc Bitoun, France Piétri-Rouxel, Sofia Benkhelifa-Ziyyat, Stéphanie Lorain

**Affiliations:** 1 Sorbonne Université, Inserm, Institut de Myologie, Centre de Recherche en Myologie, 75013 Paris, France; 2 Present Address: AFM-Téléthon, 1 rue de l’Internationale, BP59, 91002 Evry, France

**Keywords:** Gene therapy, Genetic vectors

## Abstract

Duchenne muscular dystrophy is a severe neuromuscular disease causing a progressive muscle wasting due to mutations in the *DMD* gene that lead to the absence of dystrophin protein. Adeno-associated virus (AAV)-based therapies aiming to restore dystrophin in muscles, by either exon skipping or microdystrophin expression, are very promising. However, the absence of dystrophin induces cellular perturbations that hinder AAV therapy efficiency. We focused here on the impact of the necrosis-regeneration process leading to nuclear centralization in myofiber, a common feature of human myopathies, on AAV transduction efficiency. We generated centronucleated myofibers by cardiotoxin injection in wild-type muscles prior to AAV injection. Intramuscular injections of AAV1 vectors show that transgene expression was drastically reduced in regenerated muscles, even when the AAV injection occurred 10 months post-regeneration. We show also that AAV genomes were not lost from cardiotoxin regenerated muscle and were properly localised in the myofiber nuclei but were less transcribed leading to muscle transduction defect. A similar defect was observed in muscles of the DMD mouse model *mdx*. Therefore, the regeneration process per se could participate to the AAV-mediated transduction defect observed in dystrophic muscles which may limit AAV-based therapies.

## Introduction

The dystrophinopathies are pathologies caused by mutations in the *DMD* gene that encodes the subsarcolemmal protein dystrophin. This protein is absent in Duchenne muscular dystrophy (DMD) while it is present but qualitatively and/or quantitatively altered in the less severe Becker muscular dystrophy. The lack of dystrophin in DMD muscles is the initiating event leading to many secondary changes including disruption of the sarcolemma, accumulation of endomysial fibrosis and fatty tissue^1,2^, and increased excretion of exosomes^3^. Moreover, the dystrophic muscles weakened by the dystrophin absence undergo cycles of necrosis and regeneration that lead to continuous formation of new myofibers characterized by centrally located nuclei, a hallmark of dystrophic muscles^2^.

The large size of the dystrophin cDNA prevents its packaging in a viral vector to re-express the full-length dystrophin in a deficient muscle. However, given that dystrophin tolerates large internal deletions^3^, two therapeutic strategies have been developed for DMD: the transfer of truncated cDNAs expressing functional microdystrophins, and the targeted exon(s) skipping strategy using antisense sequences converting an out-of-frame mutation into an in-frame mutation also leading to express a shorter but partially functional dystrophin. Microdystrophin expression and U7 snRNA-mediated exon skipping have shown promising results using adeno-associated virus (AAV) vectors which allow efficient transfer of the therapeutic molecules into skeletal muscles^4,5^. In particular, one-shot treatment with AAVs expressing a U7 snRNA (AAV1-U7) is sufficient to elicit substantial levels of restored dystrophin associated with a remarkable improvement of the muscle force in DMD murine models^6–8^ and in the canine GRMD model^4,9,10^. AAV9-U7 approach is also developed in mouse and primate to skip one copy of duplicated exon 2 resulting in this case to full-length dystrophin transcript and production of a wild-type protein^11,12^. These studies have laid the foundations of clinical trials currently in progress in humans (AAV-microdystrophin: NCT03368742, NCT03375164, NCT03362502; AAV9-U7: NCT04240314). However, efficacy of exon skipping by AAV1-U7 is drastically lower in muscles of the dystrophin-deficient *mdx* mouse model compared to wild-type^9,13^ and dystrophin levels decrease with time after AAV injection in the skeletal muscles from the GRMD dog^9^ and from the severely dystrophic dystrophin/utrophin double knockout (dKO) mouse model^9,13^. These data demonstrate that the dystrophic context influences the AAV-mediated transgene expression and the subsequent therapeutic benefit. Accordingly, the potential impacts on the AAV-mediated muscle transduction of the dystrophic features need to be more precisely characterized. AAV gene therapy vectors package a transgene under the control of a promoter flanked by the inverted terminal repeats (ITRs) of AAV serotype 2 (AAV2)^14^. Thereafter, these therapeutic AAVs reach cell nuclei by multiple-step events including receptor-mediated endocytosis, intracellular trafficking through the endosomal system and nucleus entry in which the vector uncoats and synthesizes the second-strand of its genome to express the transgene^15^. Thus, the therapeutic vector fate and transgene expression efficiency may be affected in regenerated muscle characterized by abnormal position of the nuclei at the centre of the fibres. In order to investigate the impact of the regeneration process independently from other features linked to dystrophin absence, we compared AAV1 transduction efficiency in muscles from *mdx* mice and from wild-type mice in which regeneration was induced by cardiotoxin (CTX), a myonecrotic agent. Although AAV genomes are properly localized in nuclei, we showed that AAV-mediated transgene expression is similarly reduced in muscles from *mdx* and CTX-treated wild-type mice demonstrating that AAV transduction is modified in regenerated muscle fibres and suggesting that muscle regeneration per se is a factor of the deficiency of AAV-mediated transgene expression in dystrophic muscles.

## Results

### The morphometric features are similarly changed in muscles from CTX-treated and *mdx* mice

**Figure 1:**
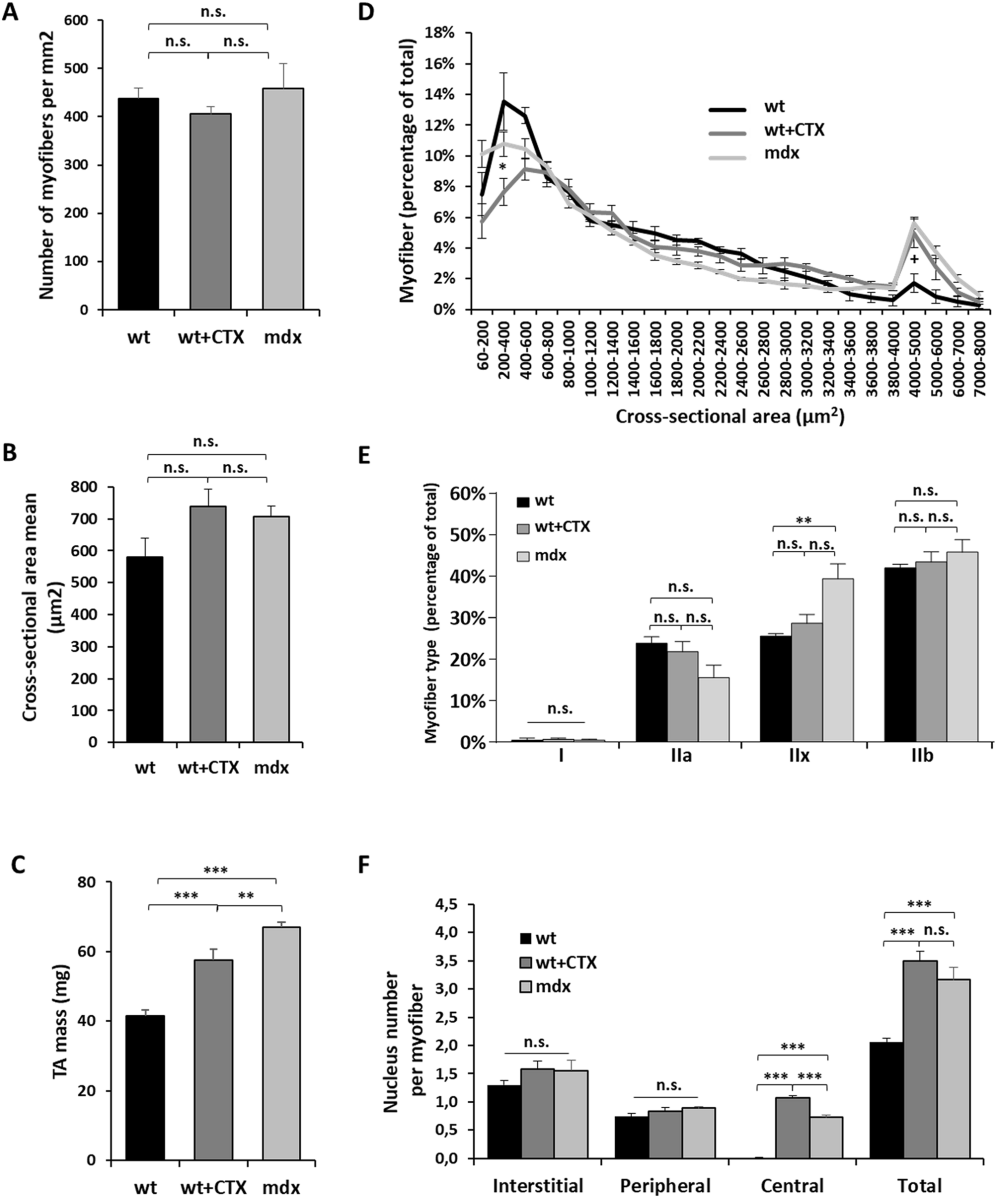
Description of cardiotoxin regenerated and *mdx* muscles. *Tibialis anterior* (TA) muscles of wild-type (wt) mice were injected or not with 0.5 nmol of cardiotoxin (CTX) to induce muscle regeneration. Six weeks after injury TAs were collected and analysed. (**A**) Number of myofibers per mm^2^ of wt, wt + CTX and *mdx* muscles. (**B**) Quantification of cross-sectional area (CSA) mean. (**C**) Muscle mass. (**D**) Distribution of myofiber CSA. (**E**) Percentage of fibers expressing I, IIa, IIx and IIb myosin heavy chain isoforms. (**F**) Counts of muscle nuclei classified into interstitial, peripheral and central localization. The data represent the mean values of four TAs per group ± SEM. n.s., non-significant, *P ≤ 0.05, **P ≤ 0.01, ***P ≤ 0.001. For D, *P ≤ 0.05 between wt + CTX group and wt or *mdx* groups, + *P* ≤ 0.05 between wt group and wt + CTX or *mdx* groups.

In order to measure AAV transduction in a regenerated muscle independently from other features of dystrophic muscles, we first induced regeneration in *Tibialis anterior* (TA) muscle from wild-type (wt) mouse by cardiotoxin (CTX) injection and compared morphometric parameters six weeks later with values measured in muscle from the DMD mouse model *mdx*^16^. In muscles from wt mice, CTX treated wt mice (wt + CTX) and *mdx* mice, the number of myofibers per mm^2^ and the cross-sectional area mean (CSA) were identical (Fig. 1A,B). As expected^17,18^, wt + CTX and *mdx* muscles presented a mass superior to wt ones by 39% and 61% respectively (Fig. 1C). As already shown^19^, the muscle hypertrophy was correlated with an increase in the number of big fibers (superior to 4000 µm^2^) and a decrease in small ones (inferior to 400 µm^2^) in wt + CTX muscles (Fig. 1D). *Mdx* muscles presented also an increase in the number of big fibers representative of dystrophic hypertrophy. The wt and *mdx* TA muscles were almost entirely composed of fast‐twitch fibers expressing respectively 23 and 16% of type IIa, 26 and 40% of type IIx and of 42 and 46% of type IIb myosin heavy chains (MHC) isoforms with rare fibers expressing the MHC‐I isoform (Fig. 1E). No major changes were observed in wt + CTX muscles compared to wt with 23% of type IIa, 28% of type IIx, and 42% of type IIb MHC isoforms.

**Figure 2:**
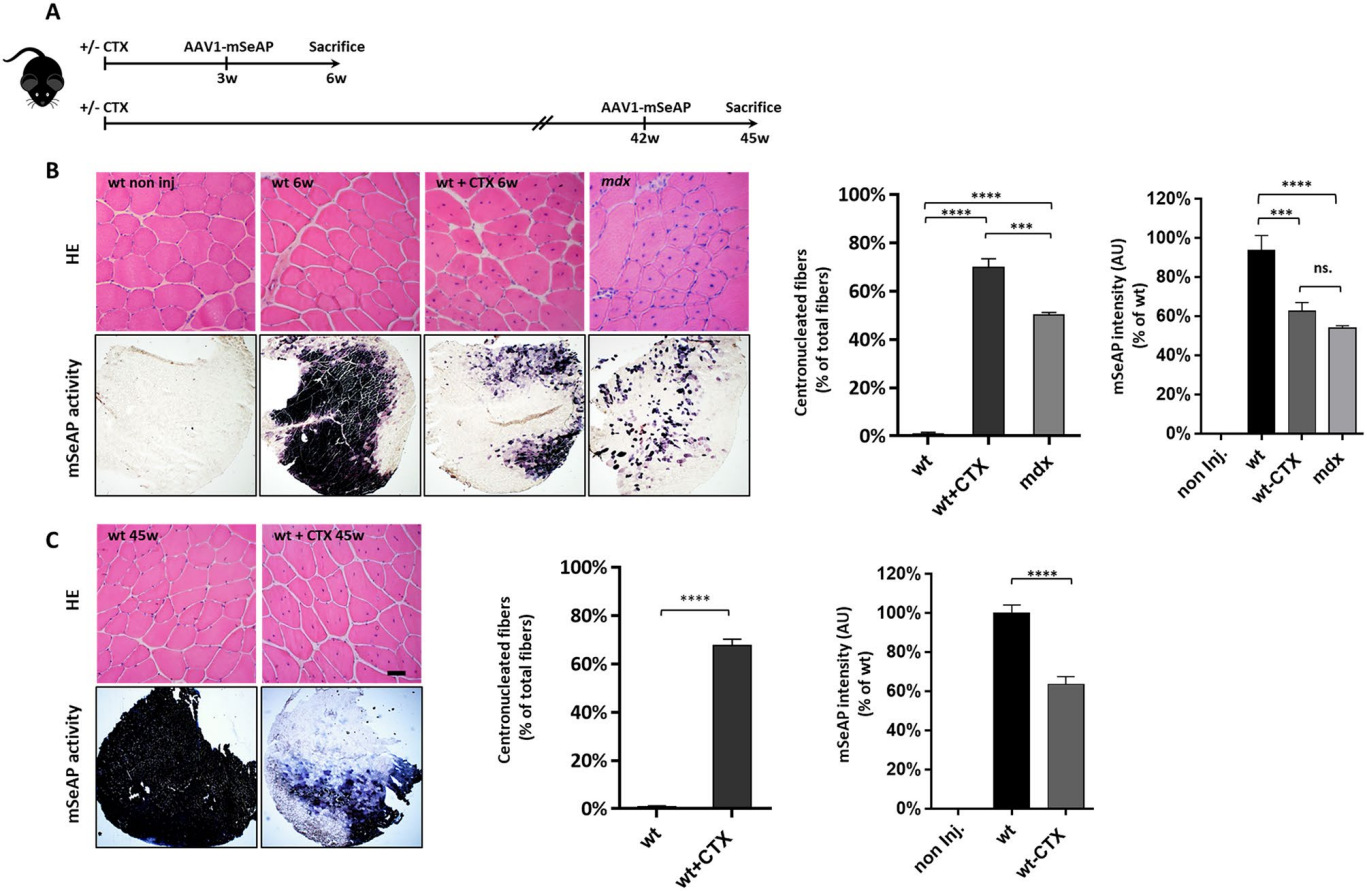
Evaluation of AAV1-mSeAP expression in cardiotoxin regenerated and *mdx* muscles. (**A**) TA muscles of wt mice were injected or not with 0.5 nmol of cardiotoxin (CTX) to induce muscle regeneration. Three weeks or 42 weeks later, the CTX muscles (wt + CTX), as well as wt muscles (wt) were injected with AAV1-mSeAP vector. Muscles were analysed 3 weeks after vector injection. TA muscles of *mdx* mice were also injected with AAV1-mSeAP vector and analysed 3 weeks after injection. Wt TAs analysed at 6 weeks = 5, wt + CTX at 6 weeks = 5, *mdx* = 6, wt at 45 weeks = 5, wt + CTX at 45 weeks = 5. (**B**) Representative (top) haematoxylin and eosin (HE) staining and (bottom) histochemical detections of mSeAP activity in TA transversal sections of non-injected wt (non inj.), wt, wt + CTX and *mdx* muscles 6 weeks after CTX injury and 3 weeks after AAV1-mSeAP injection. The quantification of the centronucleated fibers expressed as percent of total fibers as well as mSeAP activity is shown. (**C**) Representative haematoxylin and eosin (HE) staining and histochemical detections of mSeAP activity in TA transversal sections of wt and wt + CTX muscles 45 weeks after CTX injury and 3 weeks after AAV1-mSeAP injection. The quantification of the centronucleated fibers expressed as percent of total fibers as well as mSeAP activity is shown. ***p < 0.001, ****p < 0.0001. n.s., non-significant. Scale bar 100 µm.

The number of total nuclei was 1.7- and 1.5-fold higher respectively in wt + CTX and *mdx* muscles than in wt muscles (Figs. 1F, 2B and Supplementary Fig. S1). However, the number of peripheral nuclei per fiber was similar in the three conditions (around 0.8), whereas a mean of 1.1 and 0.7 central nuclei per fiber could be observed in wt + CTX and *mdx* muscles respectively that were absent in wt ones. Thus, the presence of central nuclei explains the higher number of total nuclei in wt + CTX and *mdx* muscles compared to wt. The number of interstitial nuclei per fiber was similar in the three conditions (1.5). At 3 weeks post-CTX treatment, at the moment of the AAV1-mSeAP injection, the numbers of peripheral, central and interstitial nuclei were established and no subsequent change was observed at 6, 15 and 45 weeks post-CTX when the muscles were collected for transduction efficiency analysis (Fig. S2A). It has to be noted that centrally nucleated fibers are still prevalent at 45 weeks post-CTX as recently described^20^. The number of myofibers per mm^2^ and the cross-sectional area (CSA) were also constant at all time points (Supplementary Figs. S2B,C). Overall, given that histological changes were largely similar in wt + CTX and *mdx* muscles, comparison of these two models appears pertinent in order to evaluate the specific contribution of the muscle regeneration in the impaired AAV transduction evidenced in dystrophic muscles.

### AAV1-mSeAP transduction is impaired in CTX and *mdx* muscles

To explore the impact of myofiber regeneration on AAV transduction efficiency, AAV1 vector encoding the murine secreted alkaline phosphatase (mSeAP) protein under the control of the cytomegalovirus (CMV) promoter was injected in TA muscle 3 weeks after CTX-mediated regeneration in wt mice as well as in wt and *mdx* mice (Fig. 2A). Three weeks after AAV1-mSeAP injection, and thus six weeks after CTX injection, wt + CTX and *mdx* muscles presented respectively 70% and 50% of centronucleated fibers (Fig. 2B). The mSeAP activity analysed on muscle transversal sections was dramatically reduced in wt + CTX and *mdx* muscles, respectively to 62.7 and 54.0%, compared to wt muscles. To evaluate if the AAV1-mSeAP expression defect persisted after the CTX-induced regeneration, AAV1-mSeAP vector was also injected 42 weeks after the CTX treatment (Fig. 2A). Ten months after injury (45w), we observed that myofibers still presented central nuclei and a low mSeAP activity (63.7% of wt) on muscle sections (Fig. 2C) as in muscles of 72 week-old mdx mice (Supplementary Fig. S3). In order to evaluate if the transduction defect might be linked to a side effect of the CTX treatment, regeneration was also induced in wt muscles by freeze injury and AAV was injected 4 weeks later. Three weeks after AAV injection, a low mSeAP activity was also observed in the freeze injured muscles confirming that the regeneration process per se affected the level of AAV1-mediated mSeAP expression (Supplementary Fig. S4). Altogether, these data showed that AAV1 transduction efficiency is similarly affected in regenerated muscles induced by either CTX in wt mice or dystrophin deficiency in *mdx* mice.

### AAV genomes are properly localized in nuclei in CTX regenerated and *mdx* myofibers

**Figure 3:**
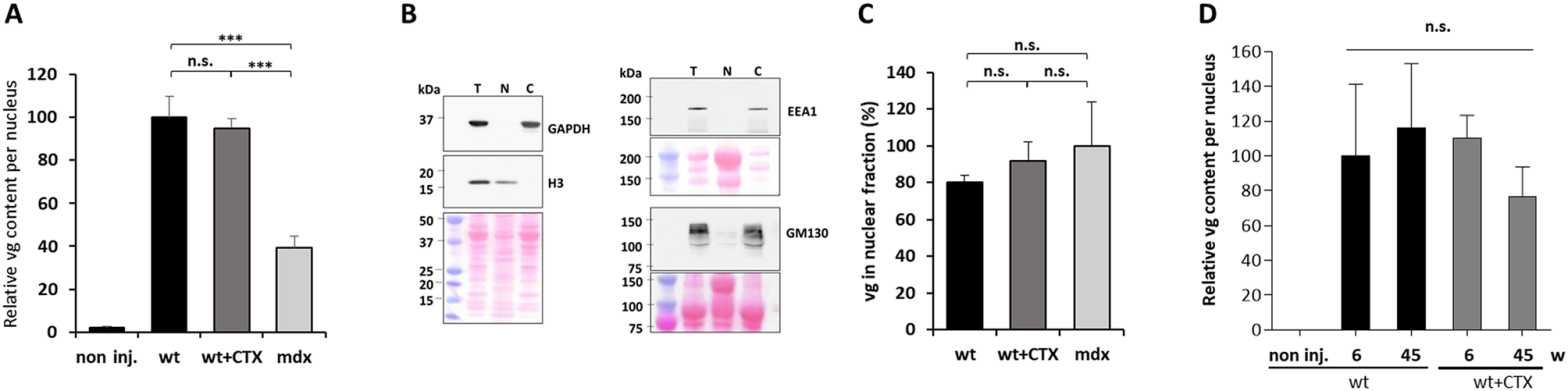
Impact of muscle regeneration on vector genome content and its intracellular localization. (**A**) Quantification of AAV genomes per nucleus by absolute qPCR in wt, wt + CTX and *mdx* muscles injected with AAV1-mSeAP vector. Three weeks after AAV injections, TAs were collected and analysed**.** Considering the higher numbers of total nuclei in wt + CTX and *mdx* muscles compared to wt muscles, the obtained data for these muscles were adjusted by a correcting factor (1.7 and 1.5, respectively). AAV genome content is expressed as the AAV genome number relative to the value obtained for wt muscles. Non inj., wt non-AAV injected TA. (**B**) Western blot analysis of total (T), nuclear (N) and cytosolic (**C**) fractions from the three muscle groups using GAPDH and H3 antibodies, confirming cytosolic and nuclear enrichments. EEA1 and GM130 antibodies detect markers of early endosomes and Golgi apparatus present in the cytosolic fractions validating the nuclear fraction purity. (**D**) AAV genomes were quantified by absolute qPCR in nuclear and total fractions from the three muscle groups and the proportion of vg present in nuclear fractions was calculated. (**D**) Quantification of AAV genomes per nucleus by absolute qPCR 10 month after regeneration. Considering the higher numbers of total nuclei in wt + CTX muscles compared to wt muscles, the obtained data for wt + CTX were adjusted by the correcting factor 1.7. AAV genome content is expressed as the AAV genome number relative to the value obtained for wt muscles (6w). Non inj., wt non-AAV injected TA. The data represent the mean values of five TAs per group ± SEM. n.s., non-significant, ***P ≤ 0.001.

Various steps of AAV-mSeAP transduction could be affected in CTX regenerated and *mdx* muscles including AAV entry into myofibers and its trafficking toward nuclei. We therefore analysed the viral genome content in the transduced muscles. We compared the vector genome (vg) contents in wt, wt + CTX and *mdx* muscles by qPCR 3 weeks after AAV1-mSeAP injection and six weeks after CTX injury. Despite the low AAV-mSeAP transduction efficiency in wt + CTX muscles, no difference was observed in the total vg copy number in the wt + CTX muscles when compared to wt muscles (Fig. 3A). Only 40% of the genomes were present in *mdx* muscles, which is coherent with the AAV genome loss from dystrophin-deficient muscles that we previously described^13,21^.

We next sought to determine the vg intracellular localization. Cell fractionation was performed from wt, wt + CTX and *mdx* muscles injected with AAV1-mSeAP for 3 weeks, and the vg contents were determined in cytoplasmic and nuclear fractions. GAPDH, the early endosome protein EEA1 and the Golgi protein GM130 were properly enriched in the cytosolic fraction whereas the H3 histone was enriched in the nuclear fraction, thereby validating the fractionation procedure (Fig. 3B). The vg proportion in the nuclear fraction was around 80–90% of total vg in the three conditions (Fig. 3C). Altogether, these results suggest a normal virus entry and viral genome maintenance in the CTX-mediated regenerated muscles. A similar amount of vg number in wt + CTX and wt muscles was also measured 45 weeks after CTX injection (Fig. 3D). Overall, viral genome entry into the muscle fibres, intracellular trafficking resulting to nuclear localization of the viral genome and maintenance of the viral genome appeared unchanged in the CTX-mediated regenerated muscles despite reduced transgene expression.

### AAV genomes are not properly transcribed in CTX regenerated and *mdx* myofibers

**Figure 4:**
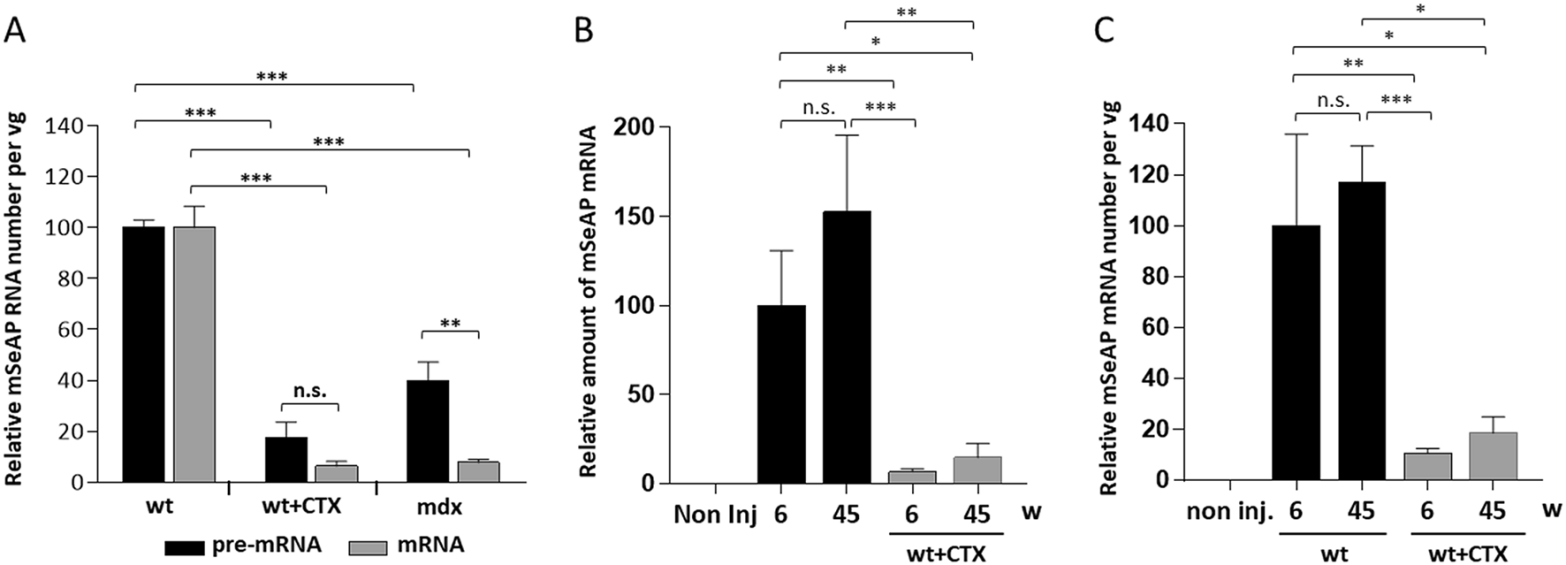
Evaluation of AAV1-mSeAP expression after muscle regeneration. (**A**) Quantification of mSeAP pre-messengers (pre-mRNA) and transcripts (mRNA) 6 weeks after CTX injury and 3 week after AAV-mSeAP injection performed by relative qPCR and normalised by the AAV genome numbers. Relative RNA number is expressed as a percentage of wt RNAs. (**B**) Quantification of mSeAP mRNA at 6 and 45 weeks post-injury by relative qPCR and expressed as a percentage of mSeAP transcripts present in wt muscles (6w). Non inj., wt non-AAV injected TA. (**C**) Quantification of mSeAP transcripts normalised by the AAV genome numbers in the corresponding muscles. Relative RNA number is expressed as a percentage of wt transcripts (6w). The data represent the mean values of minimum four TAs per group ± SEM. N.s. non-significant, *P ≤ 0.05, **P ≤ 0.01, ***P ≤ 0.001.

We then evaluated the viral genome transcription in the regenerated muscles. mSeAP pre-messenger (pre-mRNA) and messenger RNA (mRNA) numbers per vg were quantified by RT-qPCR in wt + CTX and *mdx* muscles and compared to wt muscles 3 weeks after AAV injection and six weeks after CTX injury (Fig. 4A). The amount of both RNA species per vg was drastically reduced in wt + CTX (17.4 and 6.3%) and *mdx* (40.0 and 7.8%) muscles compared to wt. Additionally, in *mdx* muscles, mSeAP mRNAs were significantly fivefold less abundant than their pre-mRNAs probably due to the mRNA instability in *mdx* muscles previously described^22^. The low amount of mSeAP pre-mRNA and transcript was also quantified at 45 weeks (14.8 and 20.4%) after CTX injury compared to wt (Fig. 4B and Supplementary Fig. S5). This low level cannot be explained by the higher muscle mass gain observed in the CTX muscles, + 24.2% at 6 weeks and + 41.4% at 45 weeks compared to wt muscles (Supplementary Fig. S2D) that would have diluted the vg content in the muscle volume by maximum a two-fold factor. When expressed per vg, as the vg contents were similar between the experimental groups, very low mSeAP transcript numbers were also observed in wt + CTX muscles with 10.6 and 18.4% at respectively 6 and 45 weeks after CTX injury compared to wt (Fig. 4C). Overall, muscle regeneration alters AAV transduction through a long-term reduction of the transgene transcription which may be involved in the transduction defect occurring in the dystrophic muscle.

### Muscle regeneration hinders AAV-mediated expression of the therapeutic U7ex23.

**Figure 5:**
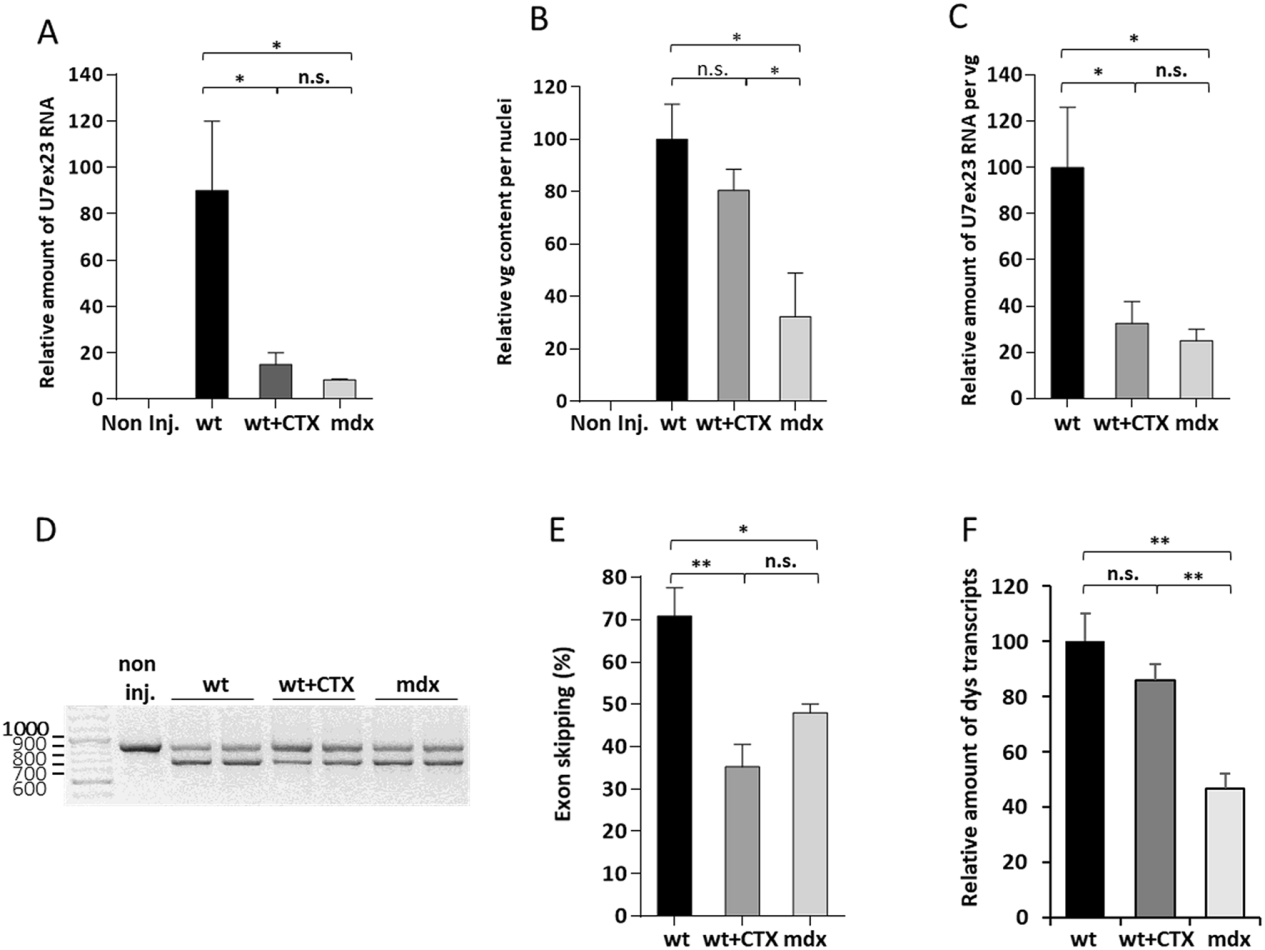
Evaluation of AAV1-U7ex23 benefit in cardiotoxin regenerated and *mdx* muscles. Wt, wt + CTX and *mdx* TAs were injected with AAV1-U7ex23 and the mice were sacrificed 3 weeks later. (**A**) Level of U7ex23 RNA estimated by relative qPCR and expressed as a percentage of U7ex23 present in wt muscles. Non inj., wt non-AAV injected TA. (**B**) Quantification of AAV genomes per nucleus by absolute qPCR in wt, wt + CTX and *mdx* muscles injected with AAV- U7ex23 vector and corrected by the correcting factors. AAV genome content is expressed as the AAV genome number relative to the value obtained for wt muscles. (**C**) U7ex23 RNA amount normalised by the AAV genome numbers and expressed as a percentage of wt RNAs. (**D**) Level of exon 23 skipping estimated by nested RT-PCR. The 901 bp PCR product corresponds to full-length dystrophin transcripts whereas the 688 bp product corresponds to transcripts lacking exon 23. (**E**) Quantification of exon 23 skipping by relative qPCR and expressed as a percentage of total dystrophin transcripts. (**F**) Quantification of dystrophin (dys) transcripts by relative qPCR. Relative amount of dys transcripts is expressed as a percentage of wt RNAs. The data represent the mean values of four TAs per group ± SEM. Student’s t-test: n.s. non-significant, *P ≤ 0.05, **P ≤ 0.01.

To investigate the effect of regeneration on AAV1-mediated expression of a therapeutic transgene, AAV1 vector encoding the U7ex23 was injected in TA muscles of wt, wt + CTX and *mdx* 3 weeks after the CTX treatment. AAV1-U7ex23 allows efficient skipping of dystrophin exon 23 that carries a nonsense mutation in *mdx* mice. As mSeAP mRNAs, U7ex23 RNAs were less abundant in wt + CTX and *mdx* muscles, respectively 17.9% and 8.1% of the amount quantified in wt muscles, showing that AAV1-U7 transduction efficiency was also reduced in regenerated muscles (Fig. 5A). As expected, the vg contents were similar in wt and wt + CTX muscles, and reduced in *mdx* muscles (42% compared to wt muscles, Fig. 5B). Consequently, low number of U7ex23 RNAs per vg were observed in wt + CTX and *mdx* muscles, respectively 31.3 and 29.2% of wt muscles (Fig. 5C). The consequence of the transduction defect on therapeutic capacity was analysed by measuring the level of exon 23 skipping by nested RT-PCR (Fig. 5D) and quantified by qPCR (Fig. 5E). In wt + CTX and *mdx* TAs, exon skipping was low, with only 35 and 48% of skipped transcripts respectively, compared to 71% in wt muscles. To understand why exon skipping was not even lower in *mdx* than wt + CTX muscles considering that the vg content per nucleus was much lower in *mdx* muscles (Fig. 5B), the number of total dystrophin transcripts was investigated in the three groups. Interestingly, the number of dystrophin transcripts in treated *mdx* muscles was half of the amount observed in wt and wt + CTX muscles (Fig. 5F) as already observed^23^, explaining why the low vg content observed in *mdx* muscles compared to wt + CTX muscles (Fig. 5B) led to similar exon skipping efficiency in these two conditions (Fig. 5E). Altogether, similar AAV1 transduction defects described for both mSeAP and U7ex23 transgenes in CTX regenerated and *mdx* muscles highlight that muscle regeneration limits the efficiency of AAV-based therapy.

## Discussion

The successful AAV-mediated gene therapy for DMD depends on efficient transduction of dystrophic muscles. However, dystrophin deficiency leads to intracellular alterations^2,24^ responsible for AAV genome loss^13,25^ or free radical-induced oxidative damage of AAV-derived transcripts limiting efficacy of the AAV-mediated therapies^22^. Accordingly, we previously showed that antisense oligonucleotides inducing temporary dystrophin expression at the sarcolemma prevent AAV genomes loss in *mdx* muscles and enhanced exon skipping therapy with a ten-fold increase of the protein level^21^. Our knowledge of abortive routes of AAV transduction in dystrophic context is still minimal and a better characterization of the consequences of the dystrophic-related cell alterations on muscle transduction by AAV vectors is instrumental for optimizing therapies especially by decreasing required dose of AAV. Dystrophic muscles undergo repeated necrosis regeneration cycles that could be, per se, a limiting factor for the AAV-based therapies. In the current study, we showed an impact of the regeneration process on the transduction efficiency of AAV in skeletal muscle. Our data revealed that AAV genomes displayed a proper nuclear localization in CTX regenerated and *mdx* muscles but expression of U7ex23 RNAs and mSeAP transcripts was similarly impaired, demonstrating a long-term transcriptional defect related to regeneration events which are common to the two muscles.

General features of skeletal muscle regeneration include a peak period of myofiber necrosis, followed by a progressive restoration of the tissue by neogenerated fibers that are centronucleated^26^. To analyse the contribution of the regeneration process to the defect of AAV-mediated transgene expression in DMD muscles, we focused on a comparative analysis of AAV transduction in one dystrophic and one non-dystrophic model of regenerated muscle. As expected^18,27^, both models presented muscle mass superior to wild-type that could be potentially responsible for a dilution of the viral genome content in the muscle volume. However, this dilution could be at most a two-fold factor and cannot explain the drastic reduction of AAV1-mediated mSeAP and U7ex23 transduction (5 to 18% of expressed transcripts compared to wild-type). Importantly, persistence of the molecular alterations affecting AAV-mediated expression up to 10 months in regenerated muscle still exhibiting central localisation of nuclei, points toward irreversible defects linked to the regeneration as a key factor affecting therapeutic transgene expression.

Several processes may limit AAV transduction in regenerated muscles. First, it has been shown that muscle fiber type can impact AAV1 transduction efficiency depending on the promoter used to mediate transgene expression^28^. However, we did not observe any major change of fiber type in CTX regenerated muscles compared to wild-type ones as already shown for *mdx* muscles^29^ suggesting that transcription defect in CTX regenerated and *mdx* muscles is not due to fiber type shift. Second, the transgene transcription defect observed in regenerated muscles may be due to the silencing of the CMV promoter as previously shown^30^. However, a similar defect was also observed with the therapeutic U7ex23 transgene which expression is driven by the U7 promoter. We showed that the regeneration impact is more drastic for mSeAP transgene than for U7ex23 with around 7% transcripts per viral genome of wt muscles in both *mdx* and CTX-injured muscles and 30% for U7ex23 suggesting that silencing of the CMV promoter could be partially implicated in the reduction of mSeAP transcript expression. It will be interesting to determine whether the regeneration process has a similar effect on the transcription in experiments with a promoter used in clinical trials like the MHCK7 promoter.

We and others have shown that the promoter activity of the viral ITR itself or of downstream promoters could be regulated by ITR binding transcription factors^31–35^ that are expressed differently in wt and regenerated muscles and may be involved in the limitation of transduction in regenerated muscles. Third, the transcriptional activity of the nuclei may be involved as transcriptional signatures of nuclei of *mdx* and CTX-regenerating muscles were shown to differ from those of the uninjured muscle^36,37^. In addition, it is well recognized that certain nuclei differ in transcriptional activities depending on their organisation or location in myofibers within a shared myofiber cytoplasm^36–38^. Moreover, myonuclei do not have equivalent capacity of expression of transgenes and endogenous genes along the same muscle fiber^38^. Indeed, while expressing and non-expressing nuclei are randomly organized in non-regenerated fibers in which the nuclei are dispersed at the periphery of the cell, they appear to be organized in active clusters flanked by inactive ones in regenerated fibers where centrally located nuclei are in close proximity to one another.

Otherwise, the endosomal processing of the AAV capsid was shown to be essential for vector uncoating, making the genome accessible to cellular factors involved in second-strand DNA synthesis and transcription^15,39^. Therefore, differences in endosomal routing in CTX regenerated muscles could lead to significant alterations of AAV processing. In spite of AAV genomes properly located in nuclei in *mdx* and CTX regenerated muscles, there is no evidence that they are efficiently released from the vector capsids and converted into the double-stranded form capable of transgene expression. Further studies with self-complementarity AAV vector in regenerated muscles should help to decipher contributions of the vector uncoating and second-strand DNA synthesis in the transcription defect. One can imagine that the transcription of viral genomes is lastingly affected in nuclei of regenerated muscles whatever the primary cause of the necrosis.

Another study previously investigated the AAV-mediated transduction efficiency in regenerating muscle^40^. AAV2 vector was shown to mediate efficiently gene transfer in notexin regenerating rat skeletal muscle compared to uninjured muscle. The use of different serotypes (AAV2 vs AAV1) could be responsible for this discrepancy. Indeed, AAV capsid structural proteins are not simply delivery vehicles for nucleic acids but also have a role in transcription of the viral genome^41^. Moreover, in addition to using AAVR, the common receptor of AAVs, AAV1 and AAV2 bind to distinct surface receptors of host cells. AAV1 binds to specific N- or O-linked sialic acid moieties while AAV2 uses heparan sulfate proteoglycan (HSPG) which is strongly expressed in young regenerating myotubes^40^ and might account for a better cell entry of AAV2 vector and a higher vg content in myonuclei compared to AAV1. Nevertheless, the main discrepancy between the two studies focused on AAV transduction in regenerated muscle is the timing of AAV injection relative to the regenerative process. While AAV2 was injected during the acute phase of regeneration (5 days after notexin injection), we injected AAV1 after muscle regeneration completion with an established localisation of the nuclei inside the myofibers. Consequently, it seems that internal cell structure reached in fully regenerated fibers hinders optimal AAV transduction and one can hypothesize that the nuclear defects play a central role. In addition, since similar transduction deficiency extends up to 10 months after CTX treatment, this limitation may be almost irreversible and may have a significant impact on the therapeutic benefit of AAV-based therapies for muscle dystrophies. In addition, two recent findings highlight the importance of better characterize the AAV fate in affected muscles which is required for development of optimal therapies for neuromuscular disorders. The first one is the demonstration that transcriptional defects limit the duration of transgene expression after AAV8-mediated Cas9 and gRNAs in *mdx* mice^25,42,43^ and the second is the decrease of AAV1-mediated transduction capacity in muscles of symptomatic animals of a mouse model of dominant centronuclear myopathy^44,45^. Therefore, as all the serotypes used today for gene therapy are based on the AAV2 genome regardless the capsid serotype, one can predict that transgene expression mediated by AAV8 or 9 would be impaired as demonstrated here for AAV1.

Finally, we highlighted a feature of the dystrophic muscle explaining the low AAV transduction efficiency in these muscles: in addition to transcript instability showed by Dupont et al*.*^22^ and AAV genome loss that we previously described^13^, we showed that the efficacy of AAV transcription is drastically affected in a sustainable manner by muscle regeneration per se. As AAV vectors are increasingly used in clinical trials for progressive dystrophies in which muscle biopsies demonstrate muscle fiber necrosis and regeneration, further studies establishing how regeneration events affecting AAV transcription may be overcome will be helpful to improve clinical use of these vectors.

## Materials and methods

### AAV vector production and animal experiments

A three-plasmid transfection protocol was used with pAAV (mSeAP)^46^ and pAAV (U7smOPT-SD23/BP22)^7^ plasmids for generation of single strand AAV1-mSeAP and AAV1-U7ex23 vectors^7,42^^.^ Vector titers were determined by qPCR and expressed as viral genomes per ml (vg/ml).

Injury was induced in *Tibialis anterior* (TA) muscles of three-month-old C57BL/6 mice by injection of 40 µl of 10 µM cardiotoxin^19^. Three to 42 weeks later, these mice were injected into the same muscles along with age and sex matched wt and *mdx* mice with 1.8E+10vg of AAV1-mSeAP or AAV1-U7ex23. Three weeks after AAV injections, muscles were collected, snap-frozen in liquid nitrogen-cooled isopentane and stored at − 80 °C. Four to five TAs were injected per group for each experiment.

All experimental protocols involving mice were performed at the Myology Research Center, Paris, France, according to the guidelines and protocols approved by the Ethical committee Charles Darwin (CEEA-005) of the French Ministry of Higher Education and Scientific Research. The present study was designed, performed, and reported according to the principles of ARRIVE (Animal Research: Reporting of In vivo Experiments) guidelines. A total of 60 mice was included in the study.

### Histological and immunofluorescence analyses

For myofiber and nucleus counting, TA cryosections of 12 µm were stained with haematoxylin and eosin. Images were acquired using an Axio Zeiss microscope and analyses were made using ImageJ software on a minimum of 4 randomly chosen images per TA with an average of 60 fibers per image.

For cross-sectional area and MHC isoform analyses, TA sections were labelled with antibodies against laminin (Dako, 1:300), MHC‐I, MHC‐IIa, MHC‐IIx and MHC‐IIb (the four latter antibodies are from DSHB, 1:10), and goat anti-rabbit secondary antibodies (Life Technologies, 1:500) and goat anti-mouse IgG1, IgM or IgG2b secondary antibodies (Thermofisher Scientific, 1:1000). Images were acquired using a Nikon AZ100 macroscope. Areas of an average of 3500 fibers per TA were measured.

Alkaline phosphatase activity was detected on TA sections as previously described^28^.

### Microscopy and image analysis

For brightfield images, a Nikon Ti2 microscope equipped with a motorized stage and coupled with CMOS DS-Ri2 Nikon camera at 10x/0.45 NA objective and 50 W Halogen Precentered Lamphouse was used. The images were acquired using NIS 5.11 software with RGB 8-bit in 5 × 5 slide Scan mode at a pixel resolution of 0.73 µm/px. Fibers were segmented with CellPose using NVIDIA GeForce RTX 3080 Ti GPU and quantified with QuPath. Images with colorimetric staining were quantified using QuPath by reducing the means of three RGB channels of the images from 8-bit to maximum Intensity (255).

### Cell fractionation and Western blot analysis

Cell fractionation from whole TAs and protein extraction were performed as described^47^. Then 25 µg of protein were loaded onto Novex NuPAGE® 4–12% Bis–Tris precast gel (Life Technologies). The membrane was probed with primary monoclonal antibodies directed against histone H3 (Cell Signaling Technology®, 1:10,000), GAPDH (Abnova, 1:15,000), EEA1 (Sigma Aldrich, 1:1,000) or GM-130 (BD Laboratories, 1:500). An incubation with sheep anti-mouse (Jackson Laboratory, 1:15,000) or goat anti-rabbit (Invitrogen, 1:10,000) horseradish peroxidase conjugated secondary antibodies allowed visualisation using Pierce™ ECL Western Blotting Substrate (Thermofisher Scientific).

### Viral genome quantification

Genomic DNA was extracted from mouse muscles and nuclear fractions using the Puregene Blood kit (Qiagen). Copy number of AAV genomes and genomic DNA were measured on 100 ng of genomic DNA by absolute quantitative PCR on a StepOnePlusTM (Applied Biosystems) using the TaqmanR Universal Master Mix (Applied Biosystems).

Primers were used to specifically amplify the viral genome sequence (F: CTCCATCACTAGGGGTTCCTTG, R: GTAGATAAGTAGCATGGC, and probe: TAGTTAATGATTAACCC) and the mouse titin gene (F: AAAACGAGCAGTGACGTGAGC, and R: TTCAGTCATGCTGCTAGCGC and probe: TGCACGGAAGCGTCTCGTCTCAGTC). As a reference sample, a pAAV plasmid and a plasmid containing the titin cDNA were tenfold serially diluted (from 10^7^ to 10^1^ copies). All genomic DNA samples were analysed in duplicates. Considering the higher numbers of total nuclei in wt + CTX and *mdx* muscles compared to wt muscles, the obtained data for these muscles were adjusted by correcting factors 1.7 and 1.5, respectively.

### RT-PCR analysis

Total RNA was isolated from mouse muscle using NucleoSpin® RNA II (Macherey–Nagel) for quantification of mSeAP pre-mRNA and mRNA, or NucleoSpin® miRNA (Macherey–Nagel) for U7ex23. Samples were purified of contaminating DNA using the DNA free kit® (Ambion). Reverse transcription (RT) was performed on 150 ng of RNA using the Superscript™ II kit (Life Technologies). mSeAP pre-mRNA, mSeAP mRNA and U7ex23 were quantified by relative quantitative PCR on a StepOnePlusTM (Applied Biosystems) using the Power SYBR® green Master Mix (ThermoFisher Scientific). mSeAP pre-mRNA primers (F: GTGCTGGCCCATCACTTTGG and R: CACATGCCGCGGGGATAAGG), mSeAP transcript primers (F: CCCTACACTGACTGCGGC and R: ATCTGCAGAATTCGCCCTTTC), U7ex23 primers (F: GGCCAAACCTCGGCTTACC and R: AGGGGTTTTCCGACCGAAG) and dysE4-E5 primers (F: GGCACTGCGGGTCTTACA and R: CATCCACTATGTCAGTGCTTCCTAT) were used to amplify mSeAP pre-mRNA, mSeAP mRNA, U7ex23 and total dystrophin transcripts. RPLPO mRNA amplification was used for normalization (F: CTCCAAGCAGATGCAGCAGA and R: ATAGCCTTGCGCATCATGGT). Non-skipped and skipped dystrophin transcripts were detected by nested PCR and quantified as described^7^.

## Statistics

Statistical analysis was performed with GraphPad Prism software (San Diego, CA). Results are expressed as means ± SEM. The significance of the difference between mean values was evaluated using one-way variance analysis (ANOVA) with a post hoc Bonferroni test in all experiments except that differences in Fig. 2C were analyzed using t-tests.

## Supplementary Information

Supplementary Figures.

## Data Availability

All data generated or analysed during this study are included in this published article and its Supplementary Information file.
