# Supplementary material for: Muscle regeneration affects Adeno Associated Virus 1 mediated transgene transcription
Source: Sci Rep. 2022 Jun 11;12:9674. doi: 10.1038/s41598-022-13405-9 (PMC9188557; doi:10.1038/s41598-022-13405-9)
Supplement: Supplementary file 1 — Supplementary Figures. [file 41598_2022_13405_MOESM1_ESM.pdf]

Supplementary Information

Muscle regeneration affects Adeno Associated Virus 1 mediated transgene transcription

Amédée Mollard[1], Cécile Peccate[1], Anne Forand[1], Julie Chassagne[1], Laura Julien[1], Pierre Meunier[1], Zoheir Guesmia[1], Thibaut Marais[1], Marc Bitoun[1], France Piétri-Rouxel[1], Sofia Benkhelifa-Ziyyat[1#*] and Stéphanie Lorain[1,2#]

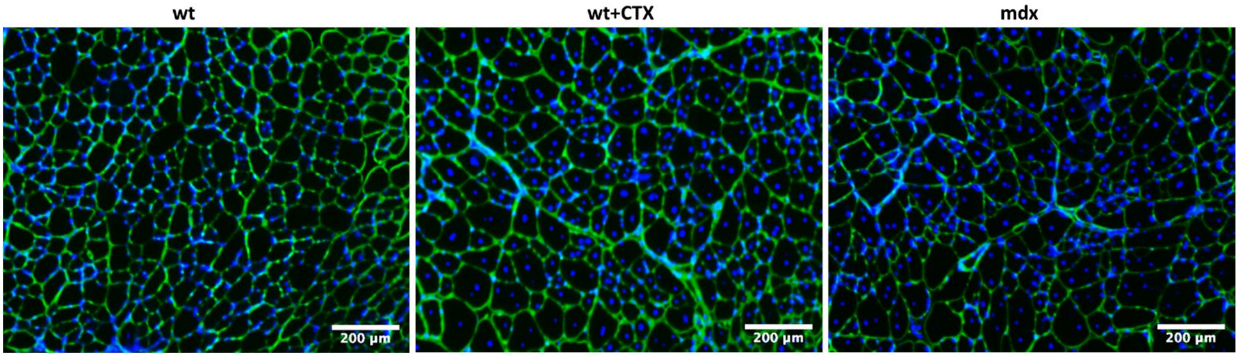

Supplementary Figure S1. Representative muscle sections of *mdx* or wt mice after regeneration induced by cardiotoxin injury.
Tibialis anterior (TA) of wild-type (wt) mice were injected or not with 0.5 nmol of cardiotoxin (CTX) to induce muscle regeneration. Six weeks after injury TAs were collected from wt, wt+CTX and *mdx* and muscle sections were labeled with DAPI to mark nuclei (blue), and anti-laminin antibody to mark the membrane (green) for the count of the number of myofibers per mm^2 of muscle, the quantification the cross-sectional area (CSA) mean of myofibers and the count of muscle nuclei classified into interstitial, peripheral and central localization as described in Figure 1.

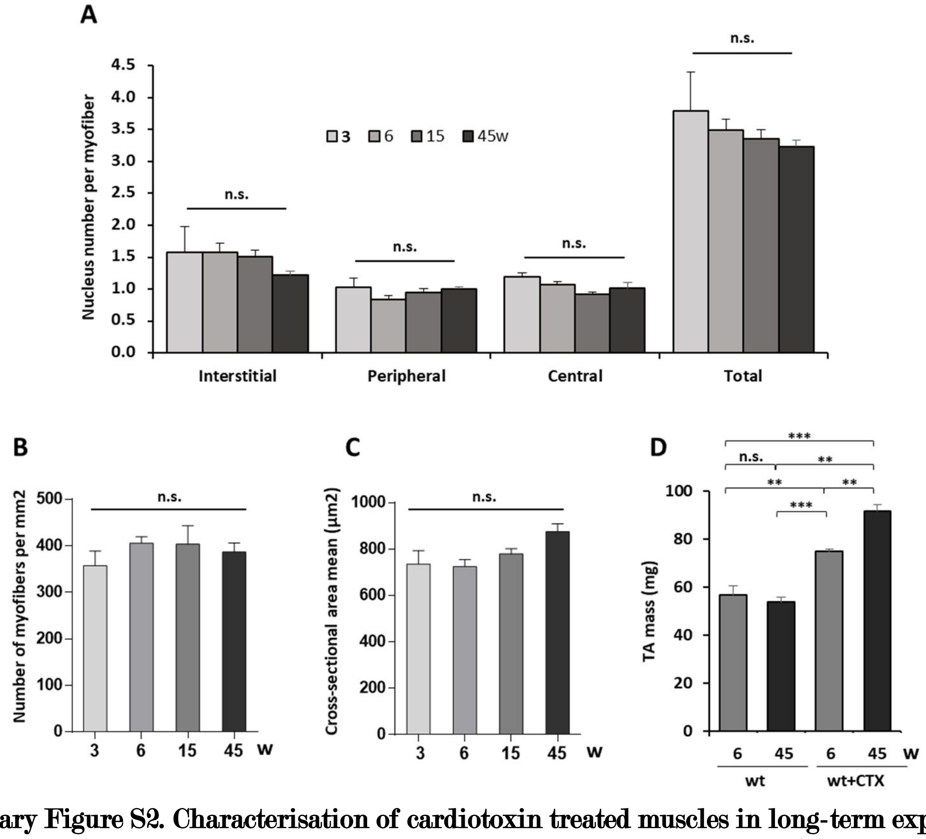

Supplementary Figure S2. Characterisation of cardiotoxin treated muscles in long-term experiment.
Wt TAs were injected (wt+CTX) or not (wt) with 0.5 nmol of cardiotoxin to induce muscle regeneration and the mice were sacrificed 3, 6, 15 or 45 weeks later. **(A)** Counts of muscle nuclei in wt+CTX muscles classified into interstitial, peripheral and central localization. **(B)** Number of myofibers per mm^2. **(C)** Quantification of cross-sectional area (CSA) mean. **(D)** Muscle mass. W, week, n.s., non-significant, ** $P \leq 0.01$, *** $P \leq 0.001$.

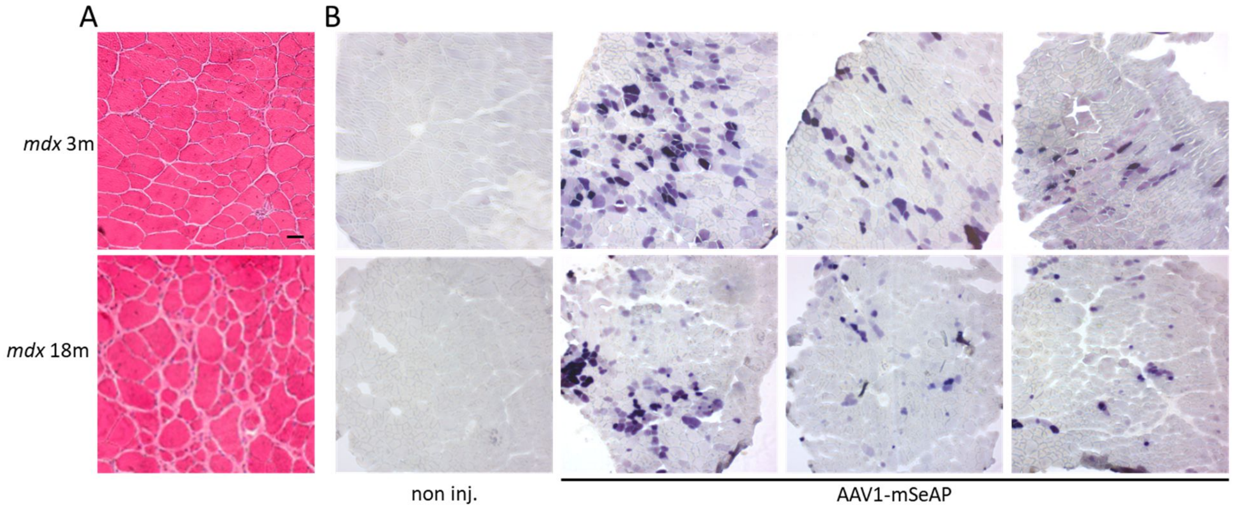

Supplementary Figure S3. Evaluation of AAV1-mSeAP expression in muscles of 3 and 18 month-old *mdx*.

(A) Representative haematoxylin and eosin (HE) staining and **(B)** histochemical detections of mSeAP activity in TA transversal sections of 3 (3m) and 18 (18m) month-old *mdx*, non-injected (non inj.) or injected with 1.8E+10vg of AAV1-mSeAP 3 weeks before muscle collection. N=3 AAV1-injected muscles for each age. Scale bar = 100 µm.

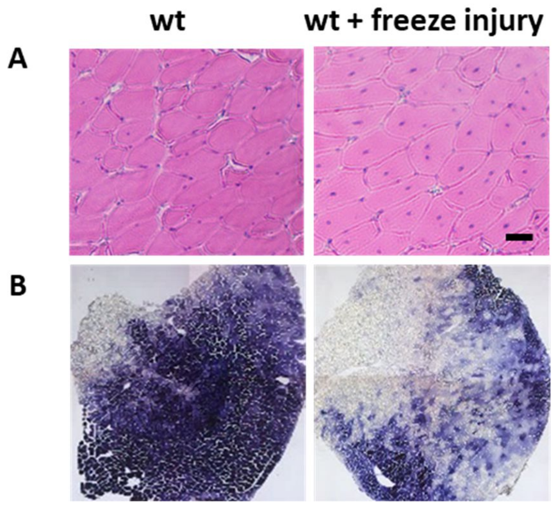

Supplementary Figure S4. Evaluation of AAV1-mSeAP expression in muscles after regeneration induced by freeze injury.

After skin incision and muscle exposition, *Tibialis anterior* (TA) muscles were frozen with three consecutive cycles of freeze-thawing by applying for 15 seconds a liquid nitrogen cooled metallic rod. The skin was sutured with 4.0 suture string and mice were kept at 37°C on a heating pad for 2 hours. Four weeks later, the same muscles (wt+freeze injury), as well as wt muscles (wt), were injected with AAV1-mSeAP vector (4 TAs per group). Muscles were analysed three weeks after AAV injections. Representative **(A)** haematoxylin and eosin (HE) staining and **(B)** histochemical detections of mSeAP activity on corresponding muscles are shown. Scale bar = 100 µm.

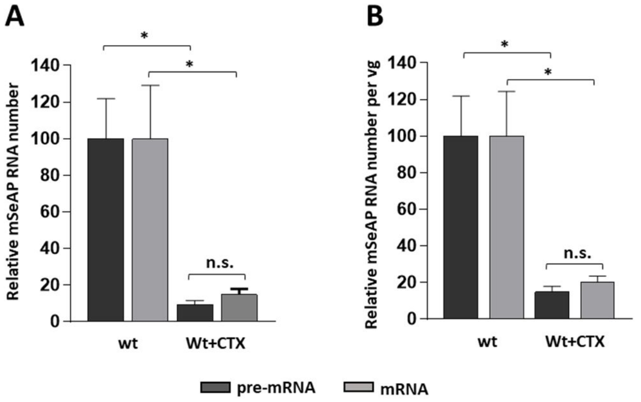

Supplementary Figure S5. Evaluation of AAV1-mSeAP expression 45 weeks after muscle regeneration. (A) Quantification of mSeAP pre-messengers (pre-mRNA) and transcripts (mRNA) 45 weeks after CTX injury and 3 week after AAV-mSeAP injection performed by relative qPCR and **(B)** normalised by the AAV genome numbers. Relative RNA number is expressed as a percentage of wt RNAs. The data represent the mean values of minimum three TAs per group ± SEM. n.s., non-significant, * $P \leq 0.05$.

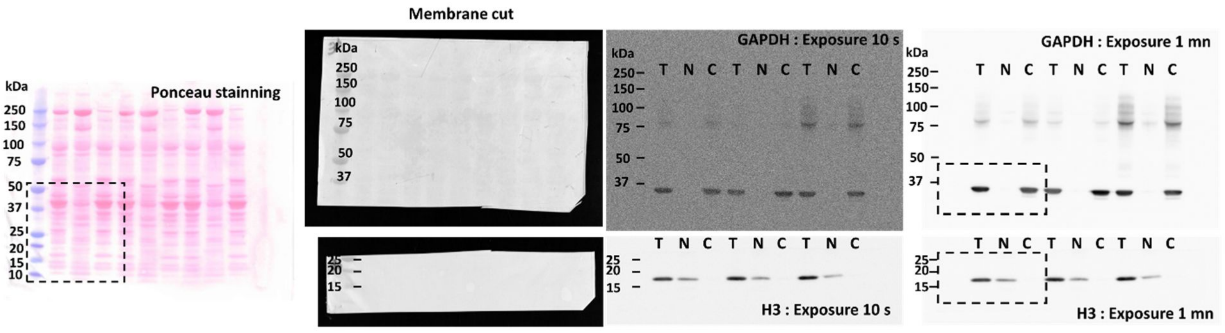

Supplementary Figure S6. Uncropped images of the Ponceau stain and GAPDH WB presented in Figure 3. For H3 WB, the same membrane was cut for hybridization to save antibodies. The presented blots are original. Total (T), nuclear (N) and cytosolic (C)

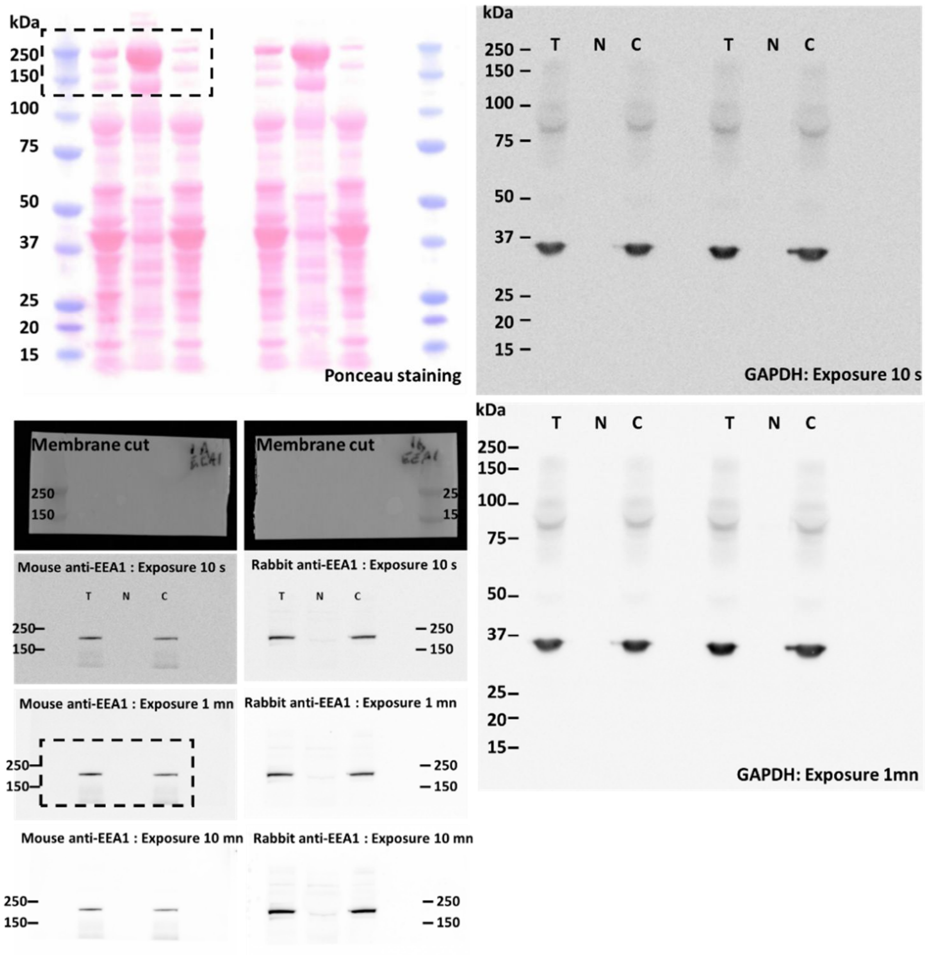

Supplementary Figure S7. Uncropped images of the Ponceau stain presented in Fig. 3 and of the corresponding GAPDH WB. For EEA1 WB presented in Fig. 3, the same membrane was cut for WB hybridization to save antibodies. The presented blots are original. Total (T), nuclear (N) and cytosolic (C)

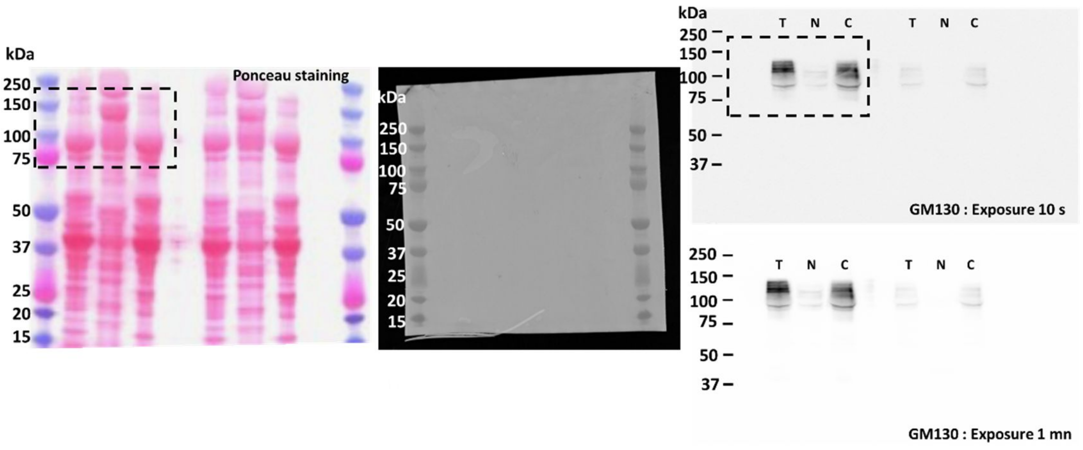

Supplementary Figure S8. The uncropped image of the Ponceau stain presented in Fig.3. For GM130 WB, the membrane was cut for hybridization to save antibodies. The presented blots are original. Total (T), nuclear (N) and cytosolic (C)

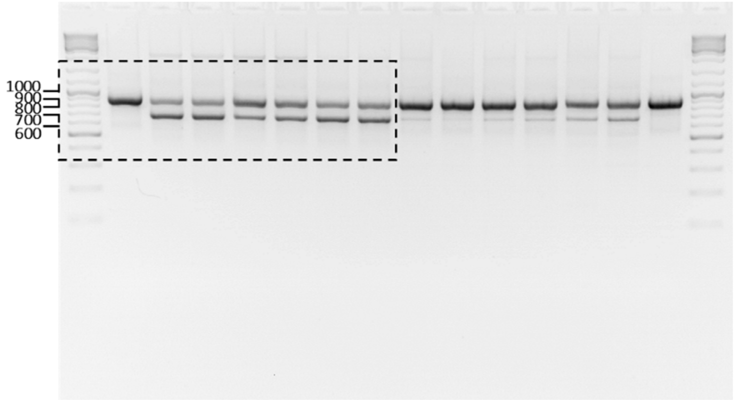

Supplementary Figure S9. Uncropped image of the gel presented in Figure 5.
